# Planetary health diet adherence, nutritional adequacy, and environmental footprints: a cross-sectional study in Turkish adults

**DOI:** 10.3389/fnut.2026.1767066

**Published:** 2026-04-02

**Authors:** Mehmetcan Kemaloğlu, Müge Yılmaz, Emine Kemaloğlu

**Affiliations:** 1 Department of Nutrition and Dietetics, Institute of Health Sciences, Erciyes University, Kayseri, Türkiye; 2 Department of Nutrition and Dietetics, Faculty of Health Sciences, Ağrı İbrahim Çeçen University, Ağrı, Türkiye; 3 Department of Nutrition and Dietetics, Faculty of Health Sciences, Erciyes University, Kayseri, Türkiye

**Keywords:** carbon footprints, diet quality, planetary health diet, sustainable nutrition, water footprints

## Abstract

**Background:**

Although the EAT–Lancet Planetary Health Diet (PHD) is recognized as a global reference model for sustainable nutrition, data on the implications of this framework in Türkiye are limited. This study aims to assess the relationship between adherence to the PHD among adults in Türkiye and both diet quality and environmental footprints.

**Methods:**

Data were collected from individuals aged 18–64 from a family health center in Ağrı province. Dietary intake was assessed using a single 24-h dietary recall. Adherence to the PHD was quantified using the Planetary Health Diet Index (PHDI), while diet quality was evaluated with the Healthy Eating Index–2020 (HEI-2020). Environmental impacts were estimated using carbon footprint (CF) and water footprint (WF) values derived from the SU-EATABLE LIFE and SHARP-ID databases.

**Results:**

Five hundred and seventy-one participants were included in the study; the mean age of the participants was 36.5 ± 12.3 years and 59.9% were female. The PHDI and HEI mean scores were 51.9 ± 12.7 and 47.5 ± 11.8, respectively. Multivariable logistic regression analysis demonstrated a strong positive relationship; participants in the highest PHDI quintile had significantly higher odds of better diet quality compared to the lowest (AOR = 9.052, 95%CI: 4.040–20.278, *p* < 0.001). Furthermore, continuous analysis confirmed that each unit increase in PHDI score was independently associated with a higher HEI score (AOR = 1.078, 95%CI: 1.053–1.103, *p* < 0.001). Among the environmental indicators, CF was inversely associated with HEI (AOR = 0.221, 95%CI: 0.125–0.393, *p* < 0.001), and WF was positively associated with HEI (AOR = 1.003, 95%CI: 1.002–1.005, *p* < 0.001).

**Conclusion:**

Higher adherence to the PHDI was linked to a better HEI score and lower environmental footprints (CF and WF). These findings highlight the importance of the PHD in promoting environmental sustainability alongside higher diet quality while reducing environmental burdens.

## 1 Introduction

Food systems are increasingly discussed as a major determinant of environmental change with far-reaching impacts on ecosystems and human health (1). Globally, food systems account for approximately 30% of greenhouse gas emissions (GHGE), use over 40% of the land area, and consume approximately 70% of freshwater resources (1–4). While adequate nutrition is one of the fundamental goals of public health, this alone does not mean that the environmental impact will be lower (5). Dietary patterns characterized by high consumption of animal-based foods place significant pressure on natural resources. This situation highlights the need for dietary approaches that can align human health with planetary boundaries (6). The need to protect our health without pushing the planetary boundaries has given rise to the need to develop dietary patterns that are both sustainable and culturally appropriate.

Therefore, the EAT-Lancet Commission proposed the Planetary Health Diet (PHD) as a global reference model, designed to promote health while staying within ecological limits (1). Subsequently, the Planetary Health Diet Index (PHDI) was developed to assess adherence to this framework (7). Evidence from studies conducted in different countries shows that higher adherence to PHD generally contributes to lower carbon footprints (CF) and water footprints (WF) (8–11). A high dietary CF, typically driven by animal-based and ultra-processed foods, not only accelerates climate change but is also directly linked to a higher risk of non-communicable diseases. Thus, assessing the CF of dietary habits is essential to understand their dual burden on planetary and human health (4). Similarly, many studies show that individuals with higher PHDI scores tend to have better overall diet quality (10, 12, 13). When all these findings are considered together, they point to the potential of dietary patterns that address both nutritional adequacy and environmental sustainability simultaneously. However, to fully understand this relationship, both parameters must be evaluated together.

In addition to environmental impacts, the analysis of sustainable nutrition models should also include comprehensive measurements of diet quality, as a low environmental footprint does not inherently ensure nutritional adequacy (14). Diets consisting primarily of nutrient-poor, refined foods may be environmentally sustainable yet associated with adverse health outcomes. Therefore, the Healthy Eating Index (HEI-2020) is widely used to assess adherence to dietary guidelines by considering both adequacy and moderation (15). Although developed in the United States, HEI has been applied in various international studies and is accepted as a suitable standard indicator for population comparisons (16). Evidence from studies conducted in Türkiye has also demonstrated significant associations between HEI scores and various health outcomes, supporting the validity of applying this index in Turkish populations (17–19). Therefore, when used in conjunction with sustainability-focused indices such as the PHDI, HEI enables a more comprehensive assessment of dietary patterns by simultaneously capturing nutritional quality and environmental impact.

Despite the increasing global interest in sustainable nutrition, studies evaluating sustainability and nutritional quality together in Türkiye remain limited. Existing studies predominantly focus on specific subpopulations, such as university students (20), and often lack simultaneous quantitative assessments of environmental footprints in adult populations (21). This limitation is particularly important given regional challenges such as water scarcity, climate sensitivity, and ongoing changes in dietary habits (22–24). In Türkiye, these changes reflect the ongoing nutrition transition characterized by a shift from the traditional Mediterranean dietary pattern toward a more Westernized diet, marked by increased consumption of energy-dense ultra-processed foods, fast food, and refined carbohydrates, alongside a reduced intake of fresh fruits, vegetables, and other plant-based foods (25, 26). Therefore, evaluating sustainable nutrition alongside diet quality and environmental indicators is essential. To our knowledge, this is the first study in Türkiye to simultaneously assess the PHDI, HEI-2020, CF, and WF in an adult population, providing a novel regional perspective from Eastern Anatolia. We aimed to examine the relationships between adherence to the EAT-Lancet dietary recommendations, measured by the PHDI, and environmental indicators (CF and WF), as well as overall diet quality. We hypothesized that greater adherence to the PHD would be associated with lower environmental footprints and higher overall diet quality.

## 2 Methods

### 2.1 Study design and participants

This cross-sectional study was conducted at a family health center in Ağrı, located in the eastern province of Türkiye, between October 2024 and March 2025. A consecutive sampling method was used, whereby all eligible individuals aged 18–64 years who attended the center for routine health services during the study period were invited to participate until the target sample size was reached. Although consecutive sampling was applied, data collection was distributed evenly across all weekdays to minimize potential selection bias related to fluctuations in participant flow or day-specific dietary variation. The minimum required sample size was calculated as 483 participants to achieve 95% statistical power with a significance level of α = 0.05 and an effect size of 0.25. To account for potential non-response or missing data, the target recruitment was increased by 10%, resulting in a planned sample of at least 532 participants (27).

**Figure 1:**
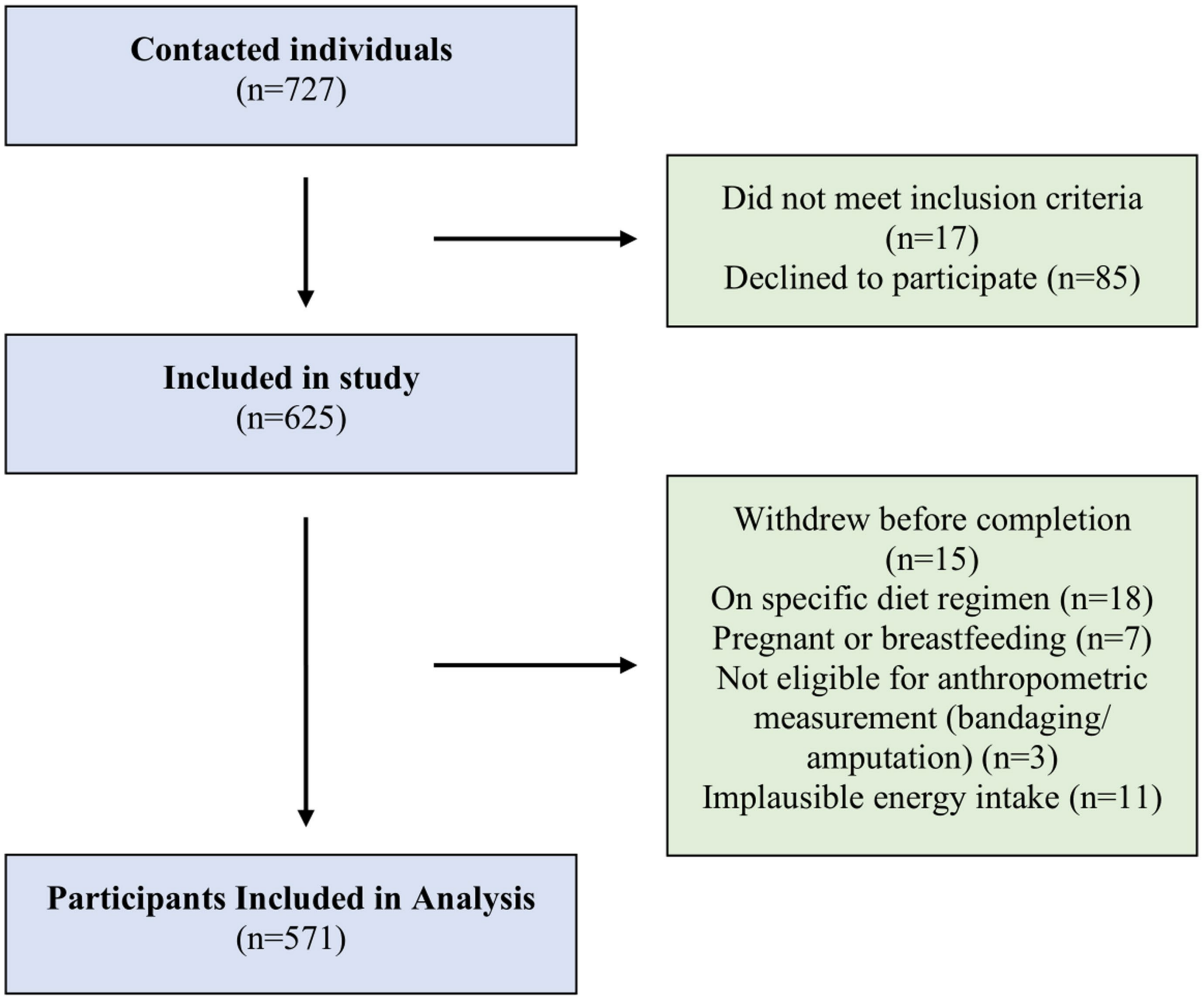
Flow diagram of the study participant selection process.

The inclusion criteria for the study were: (1) being aged 18–64 years, (2) having the ability to communicate in Turkish, (3) providing written informed consent, and (4) not following any specific therapeutic or weight-loss diet. Conversely, exclusion criteria encompassed physiological, clinical, and methodological factors. Specifically, individuals were excluded if they: (1) were pregnant or breastfeeding; (2) had physician-diagnosed conditions affecting nutritional status (e.g., eating disorders, food allergies); (3) provided missing or incomplete questionnaire responses; or (4) reported implausible energy intake values based on the 24-h dietary recall (<800 or >4,000 kcal/day for men; <500 or >3,500 kcal/day for women) (28). Participants with missing or incomplete questionnaire responses were also excluded. Trained research personnel explained the study procedures and obtained written informed consent from all participants. After applying these criteria, 571 individuals were included in the final analytic sample (Figure 1).

The study was conducted in accordance with the principles of the Declaration of Helsinki and received ethical approval from the Scientific Research Ethics Committee of Ağrı İbrahim Çeçen University (approval no. 2023/240).

### 2.2 Measures

#### 2.2.1 Demographic and socioeconomic status variables

Demographic information was collected using a structured questionnaire developed by the research team following an extensive review of previous studies in the field. In addition to age and sex, data were obtained on income level, marital status, and educational attainment. Income level was self-reported and categorized according to perceived financial status as income higher than expenses, income equal to expenses, or income less than expenses. Marital status was classified as married and separated/divorced/widowed. Educational attainment was grouped into three categories: middle school or lower, high school, and university degree or higher.

#### 2.2.2 Anthropometric assessment

Anthropometric data, including body weight and waist circumference, were measured directly by trained researchers. Height was measured to the nearest millimeter using a telescopic height gage (Seca 220, Hamburg, Germany) with the participant’s head positioned in the Frankfurt horizontal plane. Weight was assessed using a segmental body composition analyzer (Tanita SC-330, Tanita Corp., Tokyo, Japan) with participants lightly clothed (1 kg subtracted for clothing), barefoot, and after a minimum of 3 h of fasting. Body mass index (BMI, kg/m^2^) was calculated as weight in kilograms divided by height in meters squared. Waist circumference was measured at the midpoint between the iliac crest and the lower border of the tenth rib using a non-flexible tape measure while participants were standing upright (29).

#### 2.2.3 Dietary assessment

Dietary intake was assessed using a single interviewer-administered 24-h dietary recall. This method was selected because multi-day self-records often yield incomplete data, and attempting to re-contact participants for repeated recalls frequently results in high drop-out rates. All recalls were conducted face-to-face by trained researchers in standardized interviewing and probing techniques. Participants reported all foods and beverages consumed during the previous day, including cooking methods and added ingredients. Mixed dishes were disaggregated into individual components using the Standard Recipe Book, which provides standardized recipes commonly used in Türkiye (30), and each ingredient was assigned to its corresponding food group. Portion sizes were estimated using the Food and Nutrition Photograph Catalog (31), allowing participants to identify serving sizes based on visual meal samples. Dietary energy and nutrient intakes were calculated using the Nutrition Information System (BeBiS) software (The Food Code and Nutrient Database, BLS II.3, 1999, version 9.0).

#### 2.2.4 Planetary health diet index

Adherence to the EAT-Lancet recommendations was assessed using the PHDI, which reflects the extent to which individual diets align with a model intended to support both human health and environmental sustainability (7). The index consists of 16 nutritional components divided into four domains. The sufficiency domain covers foods recommended for adequate intake, including nuts and peanuts, legumes, fruits, vegetables, and whole grains. Foods expected to be consumed in optimal amounts include eggs, dairy products, fish and seafood, tubers and potatoes, and vegetable oils. Nutritional balance in vegetable consumption is captured by the proportion components, namely, the ratio of dark green vegetables and red-orange vegetables to total vegetable intake. The moderation domain represents food groups recommended for limited consumption, including red meat, poultry and substitutes, animal fats, and added sugars.

Each component is scored according to the proportion of total daily energy intake provided by the relevant food group, thereby standardizing the scores for total energy intake across participants. Components in the domains of adequacy, optimality, and moderation are assigned scores ranging from 0 to 10, while the ratio components are assigned scores ranging from 0 to 5 using previously validated scoring methods. The overall PHDI score is calculated by summing all component scores, resulting in a total score ranging from 0 to 150. Higher scores indicate higher adherence to the PHD.

#### 2.2.5 Healthy eating index-2020

Diet quality was assessed using the HEI-2020, which evaluates adherence to the 2020–2025 Dietary Guidelines for Americans (32). The index includes 13 components: nine adequacy components-total fruit, whole fruit, total vegetables, greens and beans, whole grains, dairy, total protein foods, seafood and plant proteins, and fatty acids -and four moderation components- refined grains, sodium, added sugars, and saturated fats. Component scores were calculated using energy-adjusted intake per 1,000 kcal, which enabled fair comparisons among individuals with different total energy intakes. In this scoring system, higher consumption of adequacy components contributed to higher scores, while lower intake of moderation components similarly resulted in higher scores. Component scores range from 0 to 5 or 0 to 10, and the total HEI-2020 score is obtained by summing all components, ranging from 0 to 100, with higher scores indicating better overall diet quality.

### 2.3 Assessment of the environmental indicators

This study primarily utilized the SU-EATABLE LIFE (SEL) database as the main source of environmental indicators, including CF and WF (33). CF values are expressed in kilograms of CO₂-equivalents (kg CO₂-eq) per kilogram of food, whereas WF values are expressed in liters (L) per kilogram of food.

The SEL database was selected due to its extensive coverage, transparent life cycle assessment (LCA) methodology, and accessibility for academic research. It consolidates data from peer-reviewed scientific literature, public environmental reports, and Environmental Product Declarations published up to January 2020. The database provides aggregated CF data for 323 food items and 85 food typologies, and aggregated WF data for 320 food items and 72 food typologies, all standardized per kilogram of edible portion. In SEL, system boundaries correspond to cradle-to-distribution-center, encompassing primary production, processing, packaging, and transportation, but excluding post-market stages such as retail losses, cooking, and household waste. This boundary definition aligns with the aim of the present study, which was to assess environmental impacts related to food production and distribution rather than those arising from individual consumption behaviors.

For food items not included in the SEL database, missing CF values were supplemented using the SHARP-Indicators Database (SHARP-ID) developed by Mertens et al. (34). SHARP-ID provides harmonized CF and land use (LU) values per kilogram of food as consumed, based on dietary data from four European countries (Denmark, Czech Republic, Italy, and France). System boundaries in SHARP-ID cover the full life cycle of foods-from cradle to plate-including primary production, processing, packaging, transport, retail, and home preparation. Because SEL reports environmental impacts only up to the distribution center, while SHARP-ID includes downstream stages, the system boundaries of the two datasets are not fully aligned. To minimize inconsistencies arising from these differences, SHARP-ID values were used solely for items entirely absent from SEL. In such cases, the indicators most comparable to distribution-center boundaries were selected, and the use of SHARP-ID values was clearly documented.

Both datasets rely on standardized LCA methodologies and show broadly consistent patterns across food categories (35). When multiple CF values were available for a given item, the median value was applied to reduce the influence of extreme observations and inter-study variability. Furthermore, to account for variations in total dietary volume and isolate the impact of diet quality, absolute CF and WF values were standardized per 1,000 kcal for each participant prior to all statistical analyses.

### 2.4 Statistical analysis

All analyses were performed using IBM SPSS Statistics for Windows, Version 27.0 (IBM Corp., Armonk, NY, United States). Prior to analysis, all datasets were cross-checked against the original questionnaires, and missing or erroneous entries were corrected. Participants were classified into quintiles of PHDI adherence (Q1: 8.7–40.9; Q2: 41.0–48.5; Q3: 48.6–55.4; Q4: 55.5–62.4; Q5: 62.5–90.3). HEI scores were categorized as “Poor” (≤50) and “Needs Improvement” (51–80); no participants scored above 80, and therefore the “Good” category was not used. Descriptive statistics for quantitative variables are presented as mean ± standard deviation (SD), whereas categorical variables are summarized as frequencies and percentages.

The normality of quantitative variables was evaluated using the Kolmogorov–Smirnov test. Categorical variables were examined using the chi-square test. Parametric comparisons were conducted using the Student’s *t*-test and one-way ANOVA. Non-parametrics included the Mann–Whitney U test and the Kruskal–Wallis test. To identify factors independently associated with diet quality, univariate logistic regression analyses were first performed for HEI categories. Variables with a *p*-value <0.10 in the univariate analyses were then included in the multivariable logistic regression model. Additionally, sequential multivariable logistic regression models (Models 1 to 3) were constructed to evaluate the dose–response relationship between PHDI quintiles and overall diet quality, adjusting for potential sociodemographic, nutritional, and environmental confounders. Statistical significance was set at *p* < 0.05.

## 3 Results

A total of 571 participants were included in the analysis, corresponding to a participation rate of 88.9%. Table 1 summarizes participants’ baseline characteristics according to PHDI quintiles and HEI categories. The mean age of the sample was 36.5 ± 12.3 years, and 59.9% were female. The participants’ mean BMI was 27.8 ± 5.5 kg/m^2^, waist circumference was 90.5 ± 14.0 cm, and body fat percentage was 29.8 ± 9.9%. The mean PHDI and HEI scores were 51.9 ± 12.7 and 47.5 ± 11.8, respectively. Regarding environmental indicators, the mean CF was 2.4 ± 1.3 kg CO₂-eq/1000 kcal, and the WF was 2024.2 ± 733.6 L/1000 kcal.

**Table 1:** Baseline characteristics of participants according to Planetary Health Diet Index quintiles and Healthy Eating Index-2020 categories.

Variables	Total (*n* = 571)	Quintiles of PHDI						Categories of HEI		
		Q1	Q2	Q3	Q4	Q5	*p*	Poor (≤50) (*n* = 379)	Needs improvement (51–80) (*n* = 121)	*p*
*x-* ± SD										
Age (years)	36.5 ± 12.3	39.1 ± 13.3	36.1 ± 12.7	35.6 ± 12.6	36.3 ± 11.2	35.4 ± 11.6	0.160	37.1 ± 12.8	35.6 ± 11.5	0.137
BMI (kg/m^2^)	27.8 ± 5.5	28.3 ± 6.3	28.0 ± 5.2	26.8 ± 5.0	28.0 ± 5.7	27.9 ± 4.9	0.255	27.8 ± 5.6	27.8 ± 5.3	0.869
Body fat (%)	29.8 ± 9.9	30.9 ± 10.0	30.5 ± 9.8	28.1 ± 9.3	29.9 ± 10.4	29.9 ± 9.8	0.259	29.9 ± 9.8	29.7 ± 10.0	0.823
Waist circumference (cm)	90.5 ± 14.0	91.6 ± 15.6	91.2 ± 14.4	88.2 ± 13.7	91.3 ± 12.8	90.3 ± 13.1	0.333	90.6 ± 14.1	90.4 ± 13.8	0.870
PHDI	51.9 ± 12.7	34.6 ± 6.0	44.8 ± 2.2	51.6 ± 2.0	58.7 ± 2.0	69.8 ± 6.4	**<0.001**	47.2 ± 11.2	59.2 ± 11.4	**<0.001**
HEI	47.5 ± 11.8	37.3 ± 10.6	43.3 ± 8.3	48.5 ± 8.9	51.1 ± 10.6	57.0 ± 10.2	**<0.001**	40.0 ± 7.8	59.1 ± 6.5	**<0.001**
CF (kg CO_2_ eq/1,000 kcal)	2.4 ± 1.3	2.6 ± 1.2	2.5 ± 1.1	2.8 ± 1.6	2.3 ± 1.3	2.0 ± 1.0	**<0.001**	2.5 ± 1.3	2.4 ± 1.4	0.445
WF (L/1,000 kcal)	2024.2 ± 733.6	2024.6 ± 682.5	2024.8 ± 627.1	2220.4 ± 901.3	1962.4 ± 767.4	1887.3 ± 620.5	**0.011**	1953.8 ± 688.0	2121.3 ± 783.2	**0.007**
*n* (%)										
*Gender*										
Female	342 (59.9)	70 (20.5)	72 (21.1)	67 (19.6)	64 (18.7)	69 (20.2)	0.854	209 (61.1)	133 (38.9)	0.839
Male	229 (40.1)	44 (19.2)	42 (18.3)	47 (20.5)	50 (21.8)	46 (20.1)		138 (60.3)	91 (39.7)	
*Education level*										
Middle school or below	178 (31.2)	40 (22.5)	36 (20.2)	29 (16.3)	43 (24.2)	30 (16.9)	0.061	113 (63.5)	65 (36.5)	0.303
High school	134 (23.5)	22 (16.4)	33 (24.6)	21 (15.7)	23 (17.2)	35 (26.1)		74 (55.2)	60 (44.8)	
Bachelor’s degree and higher	259 (45.4)	52 (20.1)	45 (17.4)	64 (24.7)	48 (18.5)	50 (19.3)		160 (61.8)	99 (38.2)	
*Marital status*										
Married	406 (71.1)	87 (21.4)	77 (19.0)	73 (18.0)	82 (20.2)	87 (21.4)	0.186	248 (61.1)	158 (38.9)	0.810
Separated/divorced/widowed	165 (28.9)	27 (16.4)	37 (22.4)	41 (24.8)	32 (19.4)	28 (17.0)		99 (60.0)	66 (40.0)	
*Economic status*										
Income < expense	179 (31.3)	40 (22.3)	43 (24.0)	27 (15.1)	38 (21.2)	31 (17.3)	**0.040**	107 (59.8)	72 (40.2)	0.850
Income = expense	271 (47.5)	42 (15.5)	61 (22.5)	55 (20.3)	55 (20.3)	58 (21.4)		168 (62.0)	103 (38.0)	
Income > expense	121 (21.2)	32 (26.4)	10 (8.3)	32 (26.4)	21 (17.4)	26 (21.5)		72 (59.5)	49 (40.5)	
*Smoking status*										
Never smoked	339 (59.4)	60 (17.7)	69 (20.4)	73 (21.5)	69 (20.4)	68 (20.1)	0.811	119 (60.4)	78 (39.6)	0.963
Former smoker	35 (6.1)	9 (25.7)	8 (22.9)	4 (11.4)	7 (20.0)	7 (20.0)		206 (60.8)	133 (39.2)	
Current smoker	197 (34.5)	45 (22.8)	37 (18.8)	37 (18.8)	38 (19.3)	40 (20.3)		22 (62.9)	13 (37.1)	

HEI, Healthy Eating Index; PHDI, Planetary Health Diet Index; CF, Carbon Footprint; WF, Water Footprint; Quintiles: min–max Q1: 8.7–40.9; Q2:41.0–48.5; Q3: 48.6–55.4; Q4: 55.5–62.4; Q5: 62.5–90.3; SD, standard deviation; Bold *p* values indicate statistical significance at the *p* < 0.05 level.

When PHDI quintiles were examined, age, BMI, body fat percentage, and waist circumference were not significantly different between groups (*p* > 0.05). PHDI scores increased gradually, as expected, from 34.6 ± 6.0 in Q1 to 69.8 ± 6.4 in Q5 (*p* < 0.001). Similarly, HEI scores increased across PHDI quintiles, demonstrating that higher PHDI levels were associated with better diet quality (*p* < 0.001). PHDI adherence was inversely associated with environmental footprints; both CF (*p* < 0.001) and WF (*p* = 0.011) decreased significantly from the lowest to the highest quintile.

Similarly, no significant differences were observed across HEI categories in age or anthropometric measures (*p* > 0.05). Individuals in the higher HEI category had significantly higher PHDI scores (59.2 ± 11.4 vs. 47.2 ± 11.2; p < 0.001). CF did not differ between HEI categories (*p* = 0.445), but WF was significantly higher in the higher HEI category (2121.3 ± 783.2 vs. 1953.8 ± 688.0 L/1000 kcal; *p* = 0.007).

As shown in Table 2, daily nutrient intakes differed across PHDI quintiles. Specifically, higher PHDI scores were associated with differences in several macro- and micronutrient intakes, whereas total energy and protein intakes remained similar across groups (*p* > 0.05). Higher PHDI levels were associated with lower SFA (Q1: 29.9 ± 11.7 g; Q5: 24.1 ± 10.1 g; *p* = 0.002) and higher PUFA intakes (Q1: 13.8 ± 7.1 g; Q5: 20.8 ± 10.8 g; *p* < 0.001). Similarly, fiber, vitamin C, E, B6, and folic acid intakes increased progressively across PHDI quintiles (all *p* < 0.001). Conversely, cholesterol and vitamin B12 intakes tended to decrease at higher PHDI levels (*p* < 0.001). When examining HEI categories, individuals with higher HEI scores had higher protein (84.6 ± 35.0 g vs. 78.6 ± 34.4 g; *p* = 0.027) and total fat intakes (82.2 ± 33.6 g vs. 76.3 ± 30.7 g; *p* = 0.036), while SFA intake was lower (24.2 ± 10.8 g vs. 28.4 ± 11.5 g; *p* < 0.001). PUFA intake was also significantly higher in the high HEI group (21.2 ± 11.3 g vs. 15.7 ± 8.9 g; *p* < 0.001). Furthermore, fiber, vitamin C, E, B6, folic acid, and iron intakes were significantly higher in this group (all *p* < 0.001). In contrast, calcium intake was determined to be lower in the high HEI category (623.2 ± 282.0 mg vs. 694.4 ± 323.9 mg; *p* = 0.011).

**Table 2:** Daily nutrient intakes according to Planetary Health Diet Index quintiles and Healthy Eating Index-2020 categories.

Variables	Total	Quintiles of PHDI						Categories of HEI		
		Q1	Q2	Q3	Q4	Q5	*p*	Poor (≤50) (*n* = 379)	Needs improvement (51–80) (*n* = 121)	*p*
x̄ ± SD										
Energy (kcal)	1872.0 ± 677.4	1864.4 ± 597.0	1849.7 ± 651.8	1846.8 ± 736.3	1892.0 ± 667.5	1906.6 ± 733.5	0.952	1857.0 ± 667.2	1894.4 ± 693.7	0.525
Proteins (g)	81.0 ± 34.7	80.3 ± 28.5	80.3 ± 33.7	83.2 ± 38.5	80.9 ± 36.7	80.1 ± 36.0	0.957	78.6 ± 34.4	84.6 ± 35.0	**0.027**
Fats (g)	78.6 ± 32.0	75.8 ± 26.4	76.8 ± 30.7	80.1 ± 37.7	78.8 ± 31.7	81.6 ± 32.6	0.653	76.3 ± 30.7	82.2 ± 33.6	**0.036**
SFA (g)	26.7 ± 11.4	29.9 ± 11.7	27.5 ± 11.5	26.6 ± 12.2	25.5 ± 10.9	24.1 ± 10.1	**0.002**	28.4 ± 11.5	24.2 ± 10.8	**<0.001**
MUFA (g)	27.3 ± 12.5	25.9 ± 9.7	25.3 ± 10.5	28.3 ± 15.9	27.3 ± 12.0	29.9 ± 13.2	0.079	25.7 ± 11.8	29.8 ± 13.2	**<0.001**
PUFA (g)	17.9 ± 10.3	13.8 ± 7.1	17.0 ± 9.7	18.3 ± 15.9	19.4 ± 10.4	20.8 ± 10.8	**<0.001**	15.7 ± 8.9	21.2 ± 11.3	**<0.001**
Cholesterol (mg)	397.7 ± 271.7	471.6 ± 271.9	404.4 ± 265.1	392.9 ± 281.9	389.9 ± 254.2	330.1 ± 270.3	**<0.001**	391.4 ± 273.8	407.4 ± 268.6	0.382
Dietary Fiber (g)	18.1 ± 8.4	15.0 ± 6.8	16.7 ± 7.8	17.4 ± 7.9	19.4 ± 8.4	21.8 ± 9.1	**<0.001**	16.5 ± 7.4	20.6 ± 9.2	**<0.001**
Vitamin A (mcg)	1249.1 ± 3802.7	1351.7 ± 4165.7	1616.4 ± 5844.9	1182.9 ± 3746.9	1182.3 ± 2581.7	915.0 ± 670.6	**0.018**	1274.7 ± 4305.2	1209.4 ± 2864.0	0.731
Vitamin C (mg)	97.7 ± 70.3	71.9 ± 66.6	85.1 ± 57.6	100.4 ± 74.5	109.7 ± 71.1	121.0 ± 70.6	**<0.001**	84.5 ± 64.5	118.0 ± 74.1	**<0.001**
Vitamin E (mg)	18.6 ± 10.5	14.0 ± 6.8	16.4 ± 7.2	19.7 ± 13.7	20.4 ± 9.1	22.6 ± 11.6	**<0.001**	15.7 ± 8.0	23.1 ± 12.1	**<0.001**
Vitamin B2 (mg)	1.5 ± 0.8	1.5 ± 0.9	1.6 ± 1.2	1.5 ± 0.9	1.4 ± 0.7	1.3 ± 0.5	0.164	1.5 ± 0.9	1.5 ± 0.7	0.115
Vitamin B6 (mg)	1.4 ± 0.8	1.2 ± 0.5	1.4 ± 0.8	1.5 ± 0.8	1.6 ± 0.8	1.6 ± 0.8	**<0.001**	1.3 ± 0.7	1.7 ± 0.8	**<0.001**
Folic acid (mcg)	287.5 ± 161.7	253.2 ± 158.5	281.6 ± 223.1	278.4 ± 158.6	302.6 ± 131.3	321.4 ± 108.3	**<0.001**	268.5 ± 168.7	316.9 ± 145.6	**<0.001**
Vitamin B12 (mcg)	7.0 ± 14.4	8.1 ± 16.2	8.9 ± 21.8	8.0 ± 13.9	5.5 ± 9.5	4.5 ± 4.2	**<0.001**	7.4 ± 16.4	6.4 ± 10.6	0.153
Calcium (mg)	666.5 ± 309.9	678.8 ± 310.7	699.0 ± 339.4	628.5 ± 315.4	664.1 ± 306.1	662.1 ± 275.8	0.565	694.4 ± 323.9	623.2 ± 282.0	**0.011**
Iron (mg)	10.4 ± 4.5	9.7 ± 4.6	10.2 ± 5.3	10.5 ± 4.5	10.4 ± 3.8	11.1 ± 4.3	0.066	9.6 ± 4.3	11.6 ± 4.6	**<0.001**
Zinc (mg)	10.8 ± 5.0	11.1 ± 5.0	10.9 ± 5.4	11.5 ± 5.4	10.0 ± 4.1	10.2 ± 4.9	0.213	10.6 ± 5.0	11.0 ± 4.9	0.175

HEI, Healthy Eating Index; PHDI, Planetary Health Diet Index; Quintiles: min–max Q1: 8.7–40.9; Q2:41.0–48.5; Q3: 48.6–55.4; Q4: 55.5–62.4; Q5: 62.5–90.3; PUFA, Polyunsaturated Fatty Acids (g); MUFA, Monounsaturated Fat (g); SFA, Saturated Fatty Acids (g); SD, standard deviation; Bold *p* values indicate statistical significance at the *p* < 0.05 level.

The association between Planetary Health Diet Index (PHDI) quintiles and overall diet quality (HEI) was evaluated using multivariable logistic regression, as shown in Table 3. Across all models, higher adherence to the PHDI demonstrated a significant, positive dose–response relationship with better diet quality (p for trend < 0.001). After full adjustment for sociodemographic, nutritional, and environmental covariates (Model 3), participants in the highest quintile of the PHDI (Quintile 5) exhibited significantly higher odds of having a better diet quality compared to the lowest quintile (AOR = 9.052, 95%CI: 4.040–20.278, *p* < 0.001). This robust positive association was also consistently observed in Quintile 3 and Quintile 4 (AOR = 2.715 and AOR = 4.950, respectively; *p* < 0.05).

**Table 3:** Association between Planetary Health Diet Index quintiles and Healthy Eating Index-2020.

Variables	Model 1		Model 2		Model 3	
PHDI quintiles	OR (95%CI)	*p*-value	AOR (95%CI)	*p*-value	AOR (95%CI)	*p*-value
Quintile 1	Reference	–	Reference	–	Reference	–
Quintile 2	2.048 (1.023–4.099)	0.043	2.034 (0.998–4.146)	0.051	1.227 (0.544–2.769)	0.623
Quintile 3	4.728 (2.450–9.125)	<0.001	5.025 (2.559–9.868)	<0.001	2.715 (1.253–5.886)	0.011
Quintile 4	8.448 (4.379–16.297)	<0.001	8.359 (4.256–16.417)	<0.001	4.950 (2.263–10.829)	<0.001
Quintile 5	20.271 (10.163–40.435)	<0.001	20.498 (10.138–41.444)	<0.001	9.052 (4.040–20.278)	<0.001
p for trend	–	<0.001	–	<0.001	–	<0.001

Model 1: No covariates were adjusted. Model 2: Adjusted for age, gender, education, marital status, economic status, smoking, and BMI. Model 3: Adjusted for variables in Model 2 + energy, protein, PUFA, vitamin B6, iron, CF (kg CO_2_ eq/1,000 kcal), and WF (L/1,000 kcal). OR, Odds Ratio; AOR, Adjusted Odds Ratio; CI, Confidence Interval.

Table 4 presents the univariate and multivariate logistic regression analyses examining determinants of higher HEI scores. In univariate analyses, several dietary and environmental factors including energy and protein intake, MUFA, vitamin B2, vitamin B6, iron, vitamin B12, PHDI score, CF, and WF were significantly associated with HEI scores (*p* < 0.05). In the adjusted multivariate model, energy intake (AOR = 0.999, 95%CI: 0.998–1.000; *p* = 0.006) and protein intake (AOR = 0.962, 95%CI: 0.946–0.978; *p* < 0.001) remained significant negative predictors of higher HEI scores. PUFA intake was positively associated with HEI after adjustment (AOR = 1.051, 95%CI: 1.015–1.088; *p* = 0.005). Vitamin B6 (AOR = 3.823, 95%CI: 2.104–6.946; *p* < 0.001) and iron intake (AOR = 1.270, 95%CI: 1.161–1.390; *p* < 0.001) emerged as strong independent positive predictors. Higher continuous PHDI scores were strongly and independently associated with higher HEI scores (AOR = 1.078, 95%CI: 1.053–1.103; *p* < 0.001). Environmental indicators were also highly significant in the adjusted model, with CF per 1,000 kcal showing a strong inverse association (AOR = 0.221, 95%CI: 0.125–0.393; *p* < 0.001) and WF per 1,000 kcal demonstrating a positive association (AOR = 1.003, 95%CI: 1.002–1.005; *p* < 0.001).

**Table 4:** Contrasting poor and needs improvement Healthy Eating Index categories by associated variables.

Variables	Univariate logistic regression for HEI					Multivariate logistic regression for HEI				
	β	SE	OR	CI 95%	*p*	β	SE	AOR	CI 95%	*p*
Energy	−0.002	0.001	0.998	0.996–0.999	**<0.001**	−0.001	0.000	0.999	0.998–1.000	**0.006**
Protein	−0.030	0.013	0.970	0.946–0.995	**0.019**	−0.039	0.008	0.962	0.946–0.978	**<0.001**
PUFA	0.199	0.103	1.220	0.998–1.491	0.053	0.050	0.018	1.051	1.015–1.088	**0.005**
MUFA	0.174	0.086	1.190	1.006–1.408	**0.042**	0.017	0.015	1.018	0.988–1.048	0.245
Vitamin B2	1.675	0.758	5.339	1.208–23.592	**0.027**	0.129	0.335	1.138	0.590–2.196	0.700
Vitamin B6	0.996	0.436	2.708	1.153–6.359	**0.022**	1.341	0.305	3.823	2.104–6.946	**<0.001**
Iron	0.168	0.075	1.183	1.020–1.371	**0.026**	0.239	0.046	1.270	1.161–1.390	**<0.001**
Vitamin B12	−0.097	0.055	0.907	0.814–1.011	0.079	−0.057	0.017	0.945	0.913–0.977	**0.001**
PHDI	0.070	0.014	1.072	1.043–1.102	**<0.001**	0.075	0.012	1.078	1.053–1.103	**<0.001**
CF (kg CO_2_ eq/1,000 kcal)	−0.051	0.067	0.950	0.834–1.083	0.445	−1.508	0.293	0.221	0.125–0.393	**<0.001**
WF (L/1,000 kcal)	0.000	0.000	1.000	1.000–1.001	**0.008**	0.003	0.001	1.003	1.002–1.005	**<0.001**

HEI, Healthy Eating Index; PHDI, Planetary Health Diet Index; PUFA, Polyunsaturated Fatty Acids (g); MUFA, Monounsaturated Fat (g); CF, Carbon Footprint; WF, Water Footprint; OR, Odds Ratio; AOR, Adjusted Odds Ratio; Bold *p* values indicate statistical significance at the *p* < 0.05 level.

## 4 Discussion

This study provides new evidence on the relationship between adherence to the PHDI, environmental impact indicators, and overall diet quality as measured by the HEI-2020 in an adult population in the eastern region of Türkiye. Participants who showed stronger adherence to the PHDI was associated with higher overall diet quality and more favorable nutritional profiles. Our findings revealed that higher adherence to the PHDI was consistently associated with lower CF and WF values. In contrast, higher diet quality (HEI-2020) was associated with higher WF values.

In this study, no significant differences were found between age, gender, education level, marital status, and BMI, PHDI quintiles. Findings from studies conducted in Brazil and the United States reveal that women, older adults, non-smokers, individuals with high incomes, and highly educated individuals generally show higher adherence to PHD (14, 36, 37). Likewise, Carvalho et al. (38) analyzed Portuguese national data and found that men, those with a medium level of education, and individuals with a normal BMI were more likely to have lower PHDI scores. Consistent with these findings, an assessment of data from eight Latin American countries demonstrated that higher adherence to PHD was associated with higher socioeconomic status, education, older age, and overweight or obesity (39). Furthermore, findings from the NutriNet-Santé cohort in France indicated that women, older adults, individuals with higher socioeconomic status, and those with a lower BMI had higher PHD scores (10). Studies conducted in Türkiye found that highly educated individuals, single people, and non-obese individuals had higher PHDI scores, while no significant relationship was found between gender and PHDI (20, 21). Additionally, BMI was not found to be associated with PHDI or HEI in young adults (20). Consistent with this mixed evidence, the present study identified economic status as the only sociodemographic factor differing across PHDI quintiles. This situation suggests that our participants’ dietary habits may be shaped more by regional food availability and local culinary culture than by individual demographic characteristics. In regions where food diversity or access is limited and local culinary culture is dominant, different segments of society may have similar dietary patterns. Specifically in the studied region of Eastern Türkiye, the harsh continental climate, high altitude, and prolonged winters severely restrict agricultural diversity. These geographic constraints have historically driven the local population toward animal husbandry, embedding a meat and dairy-centric culinary culture (40, 41). Consequently, the low intake of fresh produce is primarily dictated by geographic unavailability rather than individual preference. Furthermore, a recent study in Türkiye observed significantly higher PHDI adherence in coastal regions compared to Central Anatolia, further highlighting the influence of regional food environments and culinary traditions (42). Additionally, these regional factors manifest in the overall diet quality of the population, as no participant achieved a ‘Good’ HEI score (>80). The lack of high-quality dietary patterns reflects a generally poor overall diet quality. This is likely due to the combined effects of limited food diversity and the ongoing national nutrition transition.

Current findings also highlight the environmental footprints of PHDI adherence. Consistent with findings from studies, our study found that individuals with higher PHDI adherence had significantly lower CF values. A study using German DONALD cohort data found an inverse relationship between adherence to the EAT-Lancet reference diet and GHGE and LU (11). Similarly, a study analyzing data from the French NutriNet-Santé cohort also found negative relationships between adherence to an EAT-Lancet-based diet index and multiple environmental indicators (10). Studies conducted in Portugal and Israel have also associated higher PHD adherence to significantly lower GHG emissions (9, 38). Our findings support the view that higher adherence to PHD is associated with a lower CF. Furthermore, our analysis revealed a significant reduction in WF among participants with higher PHDI adherence. This demonstrates that aligning dietary patterns with planetary health guidelines optimizes water resource utilization, independent of total food intake volume. Vanham et al. (43) emphasized that the EAT-Lancet diet reduced total WF by 17–48% across countries and that this could represent significant potential for global water savings. In contrast, a large Dutch cohort study found that higher adherence to the EAT–Lancet Healthy Reference Diet was associated with higher blue water use (44). Similarly, a study conducted in Israel showed that participants who demonstrated high adherence to PHD had higher WF values (9). This difference may be partly due to the high WF content of nuts and legumes, which are encouraged in healthy diets.

High adherence to the PHD has also brought about a significant improvement in overall nutritional quality. The dietary profiles of participants who showed high adherence to the PHD are notable for their high fiber and unsaturated fat content, as well as low saturated fat and cholesterol levels. These findings are consistent with those obtained from studies conducted in France, the United Kingdom, and Türkiye (10, 21, 45). An important exception to the overall improvement in nutritional quality is vitamin B12. In our study, high PHD adherence was associated with low vitamin B12. Studies conducted in France have also reported similar decreases in B12 intake (10, 13). Given the essential role of vitamin B12 in neurological function, red blood cell formation, and DNA synthesis, insufficient intake may pose significant public health concerns, particularly among populations adopting predominantly plant-based dietary patterns. Although diets focused on sustainability tend to improve overall nutritional quality, inadequate B12 intake may increase if appropriate diet planning, food fortification, or supplementation strategies are not implemented. Therefore, public health strategies must address micronutrient needs alongside sustainability, ensuring that environmental gains do not sacrifice nutritional quality.

Our multivariable models further highlighted the robust relationship between the planetary health diet and diet quality. Sequential adjustments demonstrated that the positive dose–response association between higher PHDI quintiles and better HEI scores remained highly significant even after accounting for sociodemographic factors, individual macronutrient intakes, and environmental footprints. This indicates that the nutritional benefits of adhering to the EAT-Lancet recommendations are distinct and independent of basic demographic variations and isolated nutrient intakes. The strong positive relationship between PHDI and HEI supports the growing evidence that health-promoting dietary patterns align with sustainability goals (7). On the other hand, the observation that higher diet quality (HEI-2020) was associated with lower CF but higher WF reveals a critical environmental trade-off. This pattern suggests that improvements in nutritional quality may not translate into uniform environmental benefits across all sustainability dimensions. The increase in WF may be attributable to the greater consumption of plant-based foods such as legumes and nuts, which typically have lower carbon emissions but substantially higher irrigation requirements. Consequently, adherence to a high-HEI dietary pattern may reduce greenhouse gas emissions while simultaneously increasing water demand, underscoring the divergence between environmental indicators of dietary sustainability.

### 4.1 Strengths and limitations

This cross-sectional analysis provides important data on how adherence to the PHDI in Turkish adults relates to environmental indicators and overall diet quality. To our knowledge, this study is one of the pioneering studies in our country that examines sustainable diet models by integrating the PHDI and HEI-2020 indices with CF and WF indicators. One of the study’s strongest aspects is that anthropometric measurements were performed directly by trained researchers using standardized assessment tools. Furthermore, although participants were selected consecutively, spreading the data collection process across all days of the week helped balance potential bias arising from daily dietary variations.

However, certain limitations should be considered when interpreting the findings. The cross-sectional design of the study prevents establishing a definitive causal relationship between diet and environmental outcomes. Critically, dietary intake was recorded using only a single 24-h recall method. While this provides detailed short-term information, it may not capture intra-individual day-to-day variability or reflect long-term habitual intake, which is a major limitation in assessing the representative dietary patterns of the participants. Additionally, the fact that the data is limited to the fall and winter months (October–March) may have restricted the representation of seasonal dietary diversity. Methodologically, another limitation arises from the integration of different environmental databases. Although the SEL database was used as the primary source, the SHARP-ID database was utilized to supplement missing values. Since SEL estimates impacts up to the distribution center while SHARP-ID includes the full cradle-to-plate cycle, these discrepancies in system boundaries may have introduced uncertainties into the footprint calculations. Furthermore, the use of international databases in the absence of Türkiye-specific coefficients remains a constraint. Finally, the fact that the data was collected from a single-family health center in Eastern Türkiye limits the generalizability of the results. The specific socioeconomic and geographic structure of this region—characterized by a meat-centric culinary culture and limited agricultural diversity due to harsh climatic conditions—likely influenced the observed dietary patterns and may differ significantly from other regions in Türkiye.

### 4.2 Implications and future directions

Our findings suggest that adherence to the PHD could effectively improve diet quality and reduce CF- and WF-related environmental burdens. Particularly in today’s world where dietary habits are rapidly changing, these results support the integration of sustainability principles into national dietary guidelines. Future research should be expanded to include longitudinal studies covering different regions to clarify the causality of these relationships and better reflect seasonal effects. In this process, developing Türkiye-specific CF and WF coefficients is a critical priority to increase the accuracy of environmental analyses. Finally, while considering sustainability goals, it is of great importance to ensure that recommendations are nutritionally safe and culturally appropriate, especially for groups at risk of micronutrient deficiencies.

## 5 Conclusion

In the adult sample in Türkiye, high adherence to the PHD was found to be associated with better diet quality and both lower CF and WF values. These findings confirm the potential of healthy eating patterns to be associated with a lower environmental burden, though the magnitude of this effect varies depending on the specific environmental indicator and dietary index used. Our study provides pioneering data supporting the integration of sustainability dimensions into nutrition policies and assessments in Türkiye. In the future, comprehensive and longitudinal studies with national representation are needed to develop strategies that are both nutritionally adequate and sustainable.

## Data Availability

The raw data supporting the conclusions of this article will be made available by the authors, without undue reservation.
